# Effects of the Government Response and Community Mobility on the COVID-19 Pandemic in Southeast Asia

**DOI:** 10.3390/healthcare10102003

**Published:** 2022-10-11

**Authors:** Vita Widyasari, Chiachi Bonnie Lee, Kuan-Han Lin, Atina Husnayain, Emily Chia-Yu Su, Jiun-Yi Wang

**Affiliations:** 1 Department of Healthcare Administration, Asia University, Taichung 41354, Taiwan; 2 Cluster of Public Health Science, Faculty of Medicine, Universitas Islam Indonesia, Yogyakarta 55584, Indonesia; 3 Department of Health Services Administration, China Medical University, Taichung 406040, Taiwan; 4 Graduate Institute of Biomedical Informatics, College of Medical Science and Technology, Taipei Medical University, Taipei 10675, Taiwan; 5 Clinical Big Data Research Centre, Taipei Medical University Hospital, Taipei 10675, Taiwan; 6 Department of Medical Research, China Medical University Hospital, China Medical University, Taichung 404333, Taiwan

**Keywords:** government response, community mobility, COVID-19 pandemic

## Abstract

Preventive policies and mobility restrictions are believed to work for inhibiting the growth rate of COVID-19 cases; however, their effects have rarely been assessed and quantified in Southeast Asia. We aimed to examine the effects of the government responses and community mobility on the COVID-19 pandemic in Southeast Asian countries. The study extracted data from Coronavirus Government Response Tracker, COVID-19 Community Mobility Report, and Our World in Data between 1 March and 31 December 2020. The government responses were measured by containment, health, and economic support index. The community mobility took data on movement trends at six locations. Partial least square structural equation modeling was used for bi-monthly analyses in each country. Results show that the community mobility generally followed government responses, especially the containment index. The path coefficients of government responses to community mobility ranged from −0.785 to −0.976 in March to April and −0.670 to −0.932 in May to June. The path coefficients of community mobility to the COVID-19 cases ranged from −0.058 to −0.937 in March to April and from −0.059 to −0.640 in September to October. It suggests that the first few months since the mobility restriction implemented is the optimal time to control the pandemic.

## 1. Introduction

The COVID-19 outbreak started in 31 December 2019, in Wuhan, China, increasing rapidly and spreading massively worldwide [1]. On 20 December 2020, the numbers of cases recorded as high as 76,619,099 worldwide and 1,387,879 specifically in Southeast Asia. The case fatality rate for the closest cases is 3% [2]. In the period January 2020 and 31 October 2020, the number of cases in Indonesia, the Philippines, Myanmar, Thailand, Malaysia, and Singapore were 410,088 cases, 380,729 cases, 3780 cases, 31,548 cases, and 58,015 cases with mortality rates of 3.38%, 1.90%, 2.34%, 1.56%, 0.79%, 0.05%, respectively [3]. At the end of December 2020, several countries have shown good pandemic responses as seen in the decreasing curve of the case numbers. However, some countries have not produced the same results.

The pandemic of COVID-19 is not the first occurrence in terms of respiratory-related case events. Several diseases came to the world’s attention, such as severe acute respiratory syndrome (SARS), Middle East respiratory syndrome (MERS CoV), and avian influenza viruses, including swine flu/pandemic influenza H1N1, H7N9, H5N1, and so on [4,5,6]. The transmission of these infectious diseases is closely related to viral transmission and the extraordinary nosocomial outbreaks related to travel [7,8,9]. Many factors play a role in the spread of SARS and MERS-CoV, including the legislative factor in responses to infectious diseases, central government leadership, the creation of an intergovernmental response system, the need for coordination, information and on-site responses, late diagnosis, and family care and visits [10,11,12].

On the basis of the experience of pandemics with similar strains of the virus, several preventive steps can be taken. The target of transmission prevention is divided into two categories, namely, personal and community prevention. Personal prevention is carried out with hand hygiene, respiratory etiquette, face covers, flu vaccine, daily health monitor, and environmental disinfection [13,14,15]. Meanwhile, community deterrence is carried out by social/physical distance orders; stay-at-home orders; closure of education, location, and non-essential business; public meeting bans; travel restriction with exit and/or entry screening, detection and isolation of violent cases; and tracing and quarantine [13,14,15]. Related trends of government responses and community mobility in Latin American countries [16], the Philippines, Singapore, the United States, Sweden, Morocco, and Egypt during the COVID-19 outbreak [17] have also been studied. Despite the fact that all the countries imposed restrictions at the start of the pandemic, the percentage decrease in people’s mobility varied by country. Specifically, regarding mobility data, reductions of transit stations, workplaces, and parks were important predictors of COVID-19 daily cases [18]. Furthermore, self-imposed mobility restrictions were shown to reduce mobility by up to 14%, whereas implementing a government-sanctioned policy reduced mobility by 50%. Along with the variety of mobility, government responses are believed necessary to control the spread of disease [19].

Although these preventive policies and restrictions of mobility are believed to work for inhibiting the growth rate of COVID-19 cases, their effects have rarely been assessed and quantified in many Southeast Asian countries. To examine the relationships between these factors and outcomes, evidence on the basis of using statistical measuring tools are required, instead of merely descriptions or opinions. Therefore, the study was aimed to examine and quantify the direct and indirect effects of government responses and community mobility on the spread of COVID-19 outbreaks in Southeast Asian countries.

## 2. Materials and Methods

### 2.1. Data Sources and Study Framework

**Figure 1:**
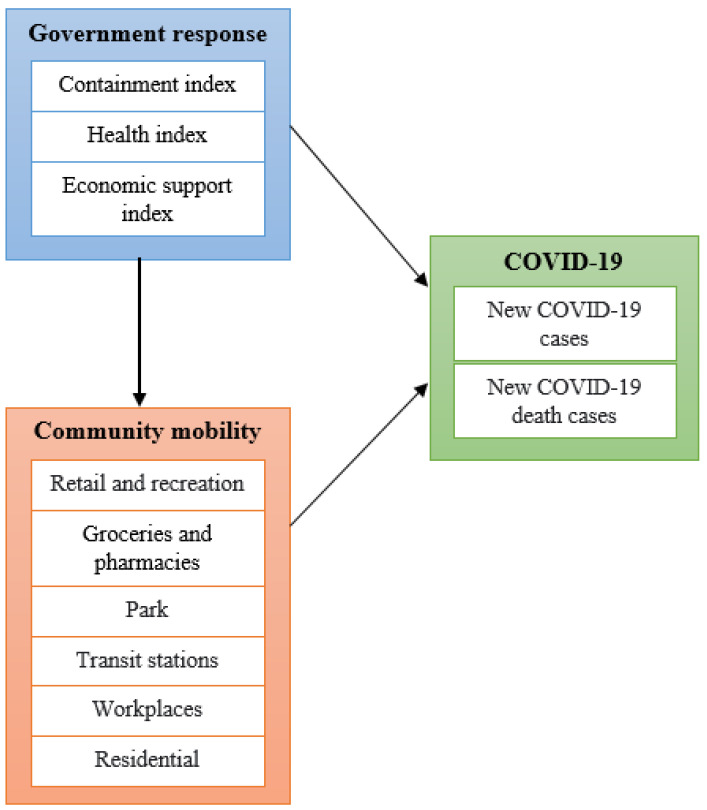
Conceptual model of the studied variables.

This study analyzed data that was observed in Southeast Asian countries from the COVID-19 Government Response Tracker (OxCGRT) [20], COVID-19 Community Mobility Report [21], and Our World in Data [22] with a cross sectional design. The study period was set between 1 March and 31 December 2020, in which many Southeast countries were facing an emergent situation of outbreak. Data on COVID-19 daily new cases and COVID-19 daily new death cases were retrieved from Our World in Data, which is a website providing data to progress against the world’s problems, including COVID-19 related data [22]. By monitoring the data day to day, the sample consisted of 306 days for analysis. Brunei and East Timor were excluded from our analysis because only sparse COVID-19 cases were observed during the study period and there is no data regarding community mobility. Figure 1 shows the conceptual model that illustrates the relationships between the studied variables. According to the study aims, the effects of government responses on community mobility and on COVID-19 cases would be assessed, as well as the effects of community mobility on COVID-19 cases. Note that the effects of government responses on COVID-19 cases could be direct or indirect (with the medication of community mobility).

### 2.2. Variables

In the conceptual model, the three latent variables are government response, community mobility, and the spread of COVID-19. Observed variables or indicators corresponding to each of the latent variables are described as follows. First, the observed variables used in the government response measure were the containment index, health index, and economic support index. A series of individual indicators for policy responses were constructed for each index, and ranges between 0 and 100 were used to estimate the value. More details are available in the website of COVID-19 Government Response Tracker [20] and briefly described as follows. The containment index was obtained from the average value of school closure policies, workplace closures, public gathering cancellation, private gathering restrictions, public transport closure, stay-at-home policy, internal movement limitations, and international travel restrictions. The health index was collected from the average value of the public policy campaign for information, testing, contact tracing, face cover, and vaccination. The economic support index was accessed from the average value of public policies regarding income support and debt or contract relief for houses. Every indicator has a varied assessment score and takes regional or national policy implementation into account. The list and operational definition of the indicators included in the three observed variables are illustrated in the Supplementary Materials (Table S1). In the economic support index indicator, for example, if a country replaced roughly half of the missing wage in all employment sectors and had a significant debt or contract relief policy, that country received a maximum score of 100. Such new policy data are released every day.

Furthermore, the community mobility refers to movement patterns across various categories of places in the community, as well as the use of public or private transportation. The report loads data on movement trends overtime at 6 locations, namely, retail and recreation (i.e., shopping malls, cafes, restaurants), groceries and pharmacies (i.e., retail markets, pharmacies, drug stores), parks (i.e., national parks, public beaches), transit stations (i.e., bus stations, train stations), workplaces, and residential areas [21]. The measurements of these observed variables were percentages compared with baseline information data. The data on this variable ranges from −100% to 100%. The baseline used for calculation was the median data from January 3 to 6 February 2020. This time period was used to determine the closest moment before COVID-19 spread widely. 

Finally, the COVID-19 spread was the third latent variable. We fetched data for daily new COVID-19 cases and those for daily new COVID-19 death cases as the observed variables.

### 2.3. Data Analysis

Considering that the whole study period was long and the relationships among variables could be sensitive to the length of study period, we divided the study period into several time windows. Along with these time windows, our analysis could illustrate the changes in trends from time to time. Considering that the maximum number of indicators was 6 among the three variables given in Figure 1, the minimum required sample size for each analysis was set at 60 based on the “Rule of 10” formula [23,24]. As a result, we divided the study period into five time windows and conducted bi-monthly analysis in each time window. Then, partial least square structural equation modeling (PLS-SEM) was used to assess relationships among variables because PLS-SEM was more flexible to accommodate small samples, abnormal data distributions, and secondary data analysis without a comprehensive substantiation of theories [25,26]. 

### 2.4. Implementation of PLS-SEM

The PLS-SEM analyses were conducted using the SmartPLS (version 3.2.8, (SmartPLS GmbH, Oststeinbek, Germany). Evaluation based on a reflective measurement model was performed before evaluating the structural model. The reflective measurement model was performed by assessing the following conditions [25]:

1. indicator loadings ≥0.70;
2. internal consistency reliability using Cronbach’s alpha and rho_A between 0.70 and 0.95, and composite reliability ≥0.70;
3. convergent validity average variance extracted (AVE) ≥0.50;
4. discriminant validity using Heterotrait-Monotrait (HTMT) ratio of correlations <0.90, cross loading, and Fornell–Larcker criterion;
5. variance inflation factor (VIF) <5.

Evaluation of the structural model was carried out by the measures of R^2^, the goodness-of-fit (*GOF*), Q^2^, and the standardized root mean square residual (SRMR). R^2^ values of 0.75, 0.50, and 0.25 are considered substantial, moderate, and weak, respectively [25]. Q^2^ value more than 0 indicate the predictive relevance of the path model [25]. The complete fit model’s *GOF* is used as a measure to ensure that the model adequately describes the empirical data. The *GOF* values range from 0 to 1, with values of 0.10, 0.25, and 0.36, which are respectively considered as small, medium, and large [27]. These values indicate the path model’s global validity. A good fit to the model indicates that the model is credible. Calculation of *GOF* used the formula:

$$GOF=\sqrt{Average\;R^{2}\times average\;AVE\;values}$$

The SRMR is a metric for estimating the model fit. When SRMR is ≤0.08, the study model is well-fitting [28]. 

The last step was to perform a bootstrapping analysis to assess the significance of the path coefficient and total effect [25]. The path coefficient discussed in this paper is the standard path coefficient. The path weights are in the range of −1 to +1 after standardization. When a value of path coefficient is close to 1, both positive and negative, it represents a powerful path. A value close to zero, on the other hand, represents a weak path. The standard path coefficient value is used for evaluation of the direct effect, while the total effect is the total value of the direct and indirect effects [23].

## 3. Results

**Figure 2:**
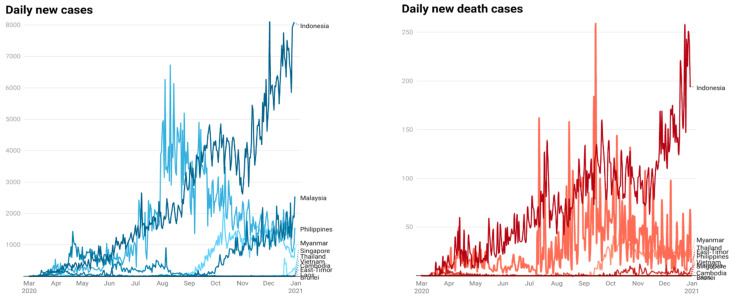
COVID-19 daily new cases and COVID-19 daily new death cases in Southeast Asian countries.

Figure 2 shows data visualization of COVID-19 daily new cases and COVID-19 daily new death cases in Southeast Asia. Table 1 shows the bi-monthly model fit and structural model evaluation for Southeast Asia. The five indicators of the reflective measurement model met the criteria well, allowing the structural model assessment to proceed. Nearly all GOF values for the Southeast Asian countries bi-monthly analysis model indicate that scientific proof adequately matches the model and has significant predictive power relative to baseline values. Considering that the SRMR was also ≤0.08, we concluded that the data matched the model well. All Q2 had a value greater than 0, indicating that it fulfills the cross-validation requirements. The value of R^2^ demonstrates that results differ depending on the window period and each country. Before presenting analytical results, several points regarding the data structure should be noticed. The calculations involving COVID-19 cases were only conducted in March to April for Laos because only few cases of COVID-19 were observed in these countries between May and December. For Singapore, the government response variable was excluded from the calculation in July to August because no policy changes happened in the three indexes in this period.

**Figure 3:**
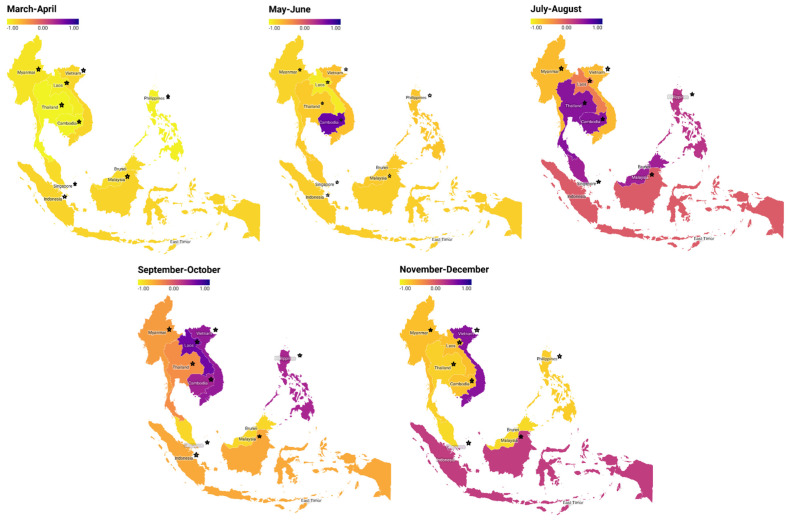
Path coefficients of government responses to community mobility in each country from the bi-monthly analysis. A star indicates a significant coefficient (*p* < 0.05). A negative value suggests that the government response could have reduced the community mobility.

Table 2 presents the detailed information of the direct effects, total and indirect effects, along with corresponding standard deviations and *p*-values. The effects of the government responses on community mobility were assessed in nine countries. In March to April, suppression effects of government responses on community mobility occurred in Southeast Asian countries with a path coefficient range between −0.785 and −0.976 (*p*-values < 0.001). Meaning, in March to April, the government responses of the nine countries could have reduced community mobility. In May to June, the obtained results are similar except for Cambodia. Other countries in Southeast Asia still showed significant suppression effects, with a path coefficient ranging between −0.670 and −0.932 (*p*-values < 0.001). Different results happened in July to August, September to October, and November to December. The directions of effects, significance, and the patterns among countries varied. Only the Philippines had a government response that had a statistically significant suppression effect on COVID-19 cases in July and August. Singapore and the Philippines continued to have a government response in September–October that had a statistically significant suppressing effect on COVID-19 cases. Only Malaysia had a government response that, in the months of November and December, statistically significantly reduced the number of COVID-19 cases. Figure 3 is presented for graphical illustration. The suppression effect (negative coefficient) is depicted in yellow in the figure, while the positive coefficient is depicted in purple. From March to June, the same trend pattern is shown for each country; the government’s response can have a suppressive effect on people’s mobility in Southeast Asian countries.

The effects of community mobility on COVID-19 cases in the nine countries were also assessed. A reduction of community mobility could suppress COVID-19 cases from March to April in Southeast Asia. Suppression effects of community mobility reduction on the COVID-19 cases occurred in these Southeast Asian countries with path coefficients ranging between −0.058 and −0.937. Small path coefficients were observed in Cambodia, Singapore, and Thailand, whereas larger and statistically significant coefficients were obtained in the other countries. Except for Vietnam, the trend of suppression effects of community mobility reduction on COVID-19 cases also recurred in September to October with path coefficients ranging between −0.059 and −0.640. In May to June, July to August, and November to December, the trend varied among these countries, without particular patterns. 

**Figure 4:**
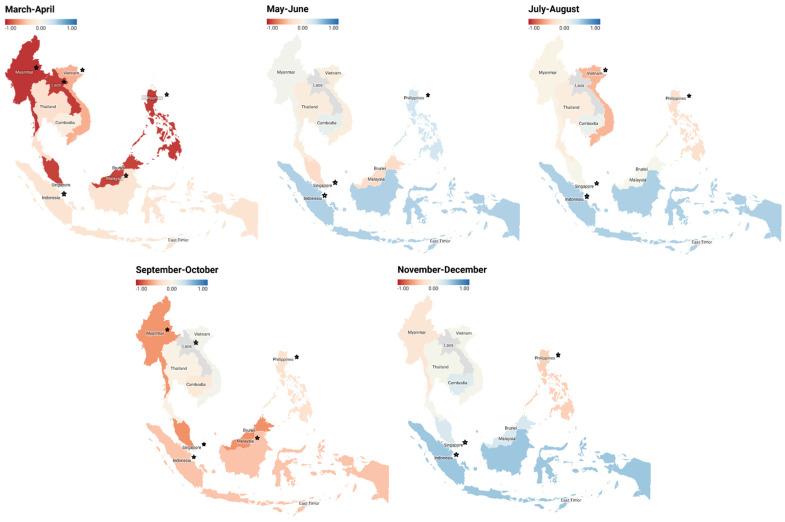
Path coefficients of community mobility to COVID-19 cases in each country from the bi-monthly analysis. A star indicates a significant coefficient (*p* < 0.05). A negative value suggests that the community mobility could suppress COVID-19 cases.

Figure 4 is presented for graphical illustration. The suppression effect (negative coefficient) is shown in red in the figure, while the positive coefficient is shown in blue. The trends between countries are aligned in March to April and September to October, indicating that community mobility can reduce the incidence of COVID-19 daily new cases and death cases; however, the effect might not be in constant.

The total effects of the government responses on COVID-19 daily new cases and death cases were assessed in all countries in Southeast Asia. From March to April, government responses positively affected the COVID-19 cases in most countries, except for Vietnam. However, no specific pattern was observed in the subsequent months.

## 4. Discussion

The present study examined and quantified the effects of government responses and community mobility on COVID-19 cases for nine countries in Southeast Asia. Based on the data retrieved from several open databases, it found that in the first several months, the government responses could have reduced the community mobility, and COVID-19 daily new cases and death cases could be suppressed by the government responses and reduced community mobility. 

The government responses had significant suppression effects on community mobility in the first several months (March to April and May to June, except for Cambodia), but no specific pattern effect was observed in the following time windows. It suggests that, in this period, the government responses could reduce community mobility in most of the countries. Several studies shows that the government played an essential role in the spread of COVID-19 cases. The self-imposed mobility was reduced by up to 14%, then after policy responses to COVID-19 announced, while the mobility could decrease up to 50% [18]. A research in 79 regions worldwide concluded that to close and restrict most places where people meet for extended periods (schools, corporations, etc.) and land boundary control are the best way to tackle COVID-19 [29]. With the implementation of disease control measures, including travel restrictions, the growth rate of COVID-19 in China became negative [30]. In terms of statistical significance and effect levels, canceling public events and implementing limitations on personal gatherings, followed by closing schools and reducing mobility patterns in various and dense locations, were valuable decisions [31]. 

Significant suppression effects of community mobility on COVID-19 daily new cases and daily new death cases were also observed. Community mobility was shown to reduce COVID-19 cases in March to April and September to October (except for Vietnam) in Southeast Asia. However, from May to June, July to August, and November to December, the trends varied among countries, showing no particular pattern. Apart from the government side, community participation by reducing mobility could also reduce the spread of COVID-19 cases. A study in 52 countries has shown that a strong correlation between reduced mobility and transmission of COVID-19 would indirectly benefit physical distancing. This implies that the lower the mobility is, the easier the physical distancing can be executed [32,33]. Note that it has been reported that mobility can affect disease dynamics with a time lag of 14.6 ± 5.6 days [34], which suggests that reducing mobility takes time to control disease spread. By contrast, Chang et al. suggested that limiting the number of people to a particular location could be more effective than reducing mobility [35].

In addition to the restriction of community mobility, it should be mentioned that vaccination is another method to suppress the spread of highly contagious diseases, though it takes a lot of research and funds to develop an effective vaccine. Based on previous epidemics, developing a single vaccine by the end of phase 2a would cost approximately $2.8 and $3.7 billion [36]. Several types of COVID-19 vaccines have also been developed and delivered to the public. These vaccines have been shown to reduce the severity of symptoms, hospitalization rates, mortalities, and the risk of recurrent infection [37,38,39], which helps people get back to normal life.

The COVID-19 outbreak can be regarded as a test for a country’s health system resilience. Countries that can handle this outbreak well are identified as having important elements of resilience. First is initiating comprehensive responses, including having a multi-ministry task force, providing training to health workers in dealing with COVID-19, cooperating in the procurement of medical devices, conducting tracing and testing accompanied by laboratory readiness, preparing isolation facilities, and frequent updating with the development of information. Second is adapting the capabilities of the healthcare system, such as providing support to health workers both financially and socially, adding health workers including empowering retirees and allocating them, setting up temporary health facilities, or transferring functions from existing facilities. Third is maintaining the healthcare system’s functions and resources by empowering community health workers to reach out to the community, having rational use guidelines to optimize existing resources, and preparing or producing health equipment. Fourth is reducing susceptibility by conducting public communications using various media, providing financial assistance for both families and businesses, as well as aggressively conducting testing and tracing [40,41].

## 5. Limitations

This research contains several limitations. First, a possible time lag exists among the COVID-19 cases, government responses, and community mobility. Diagnosis of COVID-19 at the start of the pandemic takes longer than mid-year. However, using a time lag approach a few days before the case might not be able to provide a better solution because the required time to establish a diagnosis is inconsistent. Second, community mobility data collection is limited to people who activate location history on Google Maps. Therefore, it is likely that the samples taken by Google Maps are users in their teens to adults and live in locations that are easy to get mobile phone internet services. Nonetheless, the observed community mobility data should still be representative for a large part of the population. Third, this study did not evaluate the community’s trust in the government, health resources, and the ability to procure medical equipment. Government policy and its implementation are two separate things. Government has rules, but the application is not necessarily in accordance with the written rules. The role of trust in the government makes the community willing to follow the existing rules and takes an important role in policy implementation. 

## Figures and Tables

**Table 1:** Model fit and structural model evaluation.

Evaluation Model	Mar–Apr	May–Jun	Jul–Aug	Sep–Oct	Nov–Dec
*Cambodia*					
Goodness of fit	0.667	0.473	0.376	0.377	0.503
SRMR	0.071	0.050	0.080	0.000	0.072
Q^2^ COVID-19	1.000	1.000	1.000	1.000	1.000
Q^2^ community mobility	0.562	0.455	0.489	1.000	0.546
Q^2^ government response	0.559	1.000	1.000	1.000	1.000
R^2^ COVID-19	0.001	0.045	0.037	0.135	0.050
R^2^ community mobility	0.939	0.424	0.268	0.150	0.471
*Laos*					
Goodness of fit	0.685	0.878	0.242	0.575	0.624
SRMR	0.048	0.074	0.064	0.080	0.072
Q^2^ COVID-19	1.000	-	-	-	-
Q^2^ community mobility	0.561	0.388	0.342	0.367	0.479
Q^2^ government response	0.603	1.000	1.000	1.000	1.000
R^2^ COVID-19	0.117	-	-	-	-
R^2^ community mobility	0.868	0.844	0.064	0.394	0.440
*Myanmar*					
Goodness of fit	0.745	0.540	0.503	0.692	0.517
SRMR	0.061	0.052	0.078	0.080	0.058
Q^2^ COVID-19	1.000	1.000	1.000	0.571	1.000
Q^2^ community mobility	0.481	0.422	0.316	0.537	0.441
Q^2^ government response	0.599	1.000	1.000	1.000	1.000
R^2^ COVID-19	0.400	0.003	0.136	0.860	0.116
R^2^ community mobility	0.783	0.614	0.413	0.195	0.445
*Singapore*					
Goodness of fit	0.860	0.815	0.221	0.493	0.584
SRMR	0.071	0.073	0.072	0.080	0.071
Q^2^ COVID-19	1.000	1.000	1.000	1.000	1.000
Q^2^ community mobility	0.421	0.427	0.366	0.426	0.511
Q^2^ government response	0.544	1.000	-	1.000	1.000
R^2^ COVID-19	0.779	0.593	0.058	0.360	0.355
R^2^ community mobility	0.832	0.869	-	0.173	0.377
*Vietnam*					
Goodness of fit	0.584	0.461	0.596	0.383	0.374
SRMR	0.080	0.071	0.074	0.042	0.080
Q^2^ COVID-19	1.000	1.000	1.000	1.000	1.000
Q^2^ community mobility	0.456	0.524	0.643	0.597	0.594
Q^2^ government response	0.473	1.000	0.359	1.000	0.359
R^2^ COVID-19	0.150	0.010	0.399	0.096	0.058
R^2^ community mobility	0.633	0.449	0.396	0.213	0.263
*Indonesia*					
Goodness of fit	0.783	0.757	0.311	0.490	0.549
SRMR	0.078	0.076	0.055	0.072	0.076
Q^2^ COVID-19	0.443	0.359	1.000	1.000	0.500
Q^2^ community mobility	0.417	0.537	0.608	0.490	0.361
Q^2^ government response	0.567	1.000	1.000	1.000	1.000
R^2^ COVID-19	0.792	0.760	0.203	0.253	0.663
R^2^ community mobility	0.617	0.558	0.001	0.278	0.035
*Malaysia*					
Goodness of fit	0.685	0.604	0.281	0.787	0.647
SRMR	0.080	0.080	0.078	0.080	0.073
Q^2^ COVID-19	0.344	1.000	1.000	0.488	1.000
Q^2^ community mobility	1.000	0.465	0.395	0.538	0.435
Q^2^ government response	0.426	1.000	1.000	1.000	0.437
R^2^ COVID-19	0.424	0.270	0.003	0.741	0.264
R^2^ community mobility	0.687	0.525	0.163	0.649	0.667
*Philippines*					
Goodness of fit	0.815	0.710	0.402	0.293	0.588
SRMR	0.065	0.060	0.064	0.078	0.080
Q^2^ COVID-19	0.388	1.000	1.000	1.000	1.000
Q^2^ community mobility	1.000	0.522	0.479	0.291	0.458
Q^2^ government response	1.000	1.000	1.000	1.000	0.526
R^2^ COVID-19	0.505	0.557	0.274	0.066	0.204
R^2^ community mobility	0.919	0.486	0.062	0.120	0.548
*Thailand*					
Goodness of fit	0.726	0.520	0.375	0.293	0.625
SRMR	0.046	0.053	0.074	0.061	0.067
Q^2^ COVID-19	1.000	1.000	1.000	1.000	1.000
Q^2^ community mobility	0.508	0.602	0.350	0.343	0.342
Q^2^ government response	1.000	1.000	1.000	1.000	1.000
R^2^ COVID-19	0.143	0.046	0.032	0.055	0.262
R^2^ community mobility	0.953	0.525	0.271	0.129	0.576

**Table 2:** Path coefficients of the government responses, community mobility, and COVID-19 cases in Southeast Asian countries.

Variables	Direct/Total/Indirect Effect (Standard Deviation)								
	Cambodia	Indonesia	Laos	Malaysia	Myanmar	Philippines	Singapore	Thailand	Vietnam
*March–April (n = 61)*									
Community mobility -> COVID-19 cases	−0.109 (0.461)	−0.220 (0.066) **	−0.922 (0.330) **	−0.898 (0.209) ***	−0.937 (0.162) ***	−0.918 (0.293) **	−0.058 (0.163)	−0.273 (0.530)	−0.515 (0.174) **
Government response -> COVID-19 cases	−0.097 (0.438)	0.707 (0.059) ***	−0.792 (0.381) *	−0.330 (0.201)	−0.372 (0.155) *	−0.219 (0.299)	0.829 (0.148) ***	0.107 (0.519)	−0.638 (0.143) ***
Government response -> Community mobility	−0.969 (0.009) ***	−0.785 (0.049) ***	−0.932 (0.014) ***	−0.829 (0.022) ***	−0.885 (0.017) ***	−0.959 (0.012) ***	−0.912 (0.020) ***	−0.976 (0.006) ***	−0.795 (0.037) ***
Government response -> COVID-19 cases ^†^	0.009 (0.053)	0.880 (0.019) ***	0.067 (0.122)	0.415 (0.077) ***	0.458 (0.050) ***	0.661 (0.055)	0.882 (0.023) ***	0.373 (0.090) ***	−0.228 (0.118)
Government response -> Community mobility -> COVID-19 cases ^‡^	0.106 (0.448)	0.173 (0.052) **	0.859 (0.311) **	0.745 (0.189) ***	0.830 (0.151) ***	0.880 (0.282) **	0.053 (0.150)	0.266 (0.520)	0.410 (0.139) **
*May–June (n = 61)*									
Community mobility -> COVID-19 cases	0.147 (0.127)	0.447 (0.074) ***	-	−0.309 (0.237)	0.071 (0.135)	0.327 (0.150) *	−0.672 (0.227) **	−0.122 (0.259)	−0.109 (0.153)
Government response -> COVID-19 cases	0.084 (0.105)	−0.485 (0.073) ***	-	0.251 (0.175)	0.022 (0.163)	−0.481 (0.146) **	0.104 (0.232)	0.110 (0.227)	−0.015 (0.142)
Government response -> Community mobility	0.651 (0.064) ***	−0.747 (0.033) ***	−0.919 (0.014) ***	−0.725 (0.044) ***	−0.783 (0.056) ***	−0.697 (0.047) ***	−0.932 (0.042) ***	−0.725 (0.048) ***	−0.670 (0.051) ***
Government response -> COVID-19 cases ^†^	0.180 (0.054) **	−0.820 (0.028) ***	-	0.475 (0.064) ***	−0.033 (0.120)	−0.708 (0.056) ***	0.731 (0.042) ***	0.198 (0.101) *	0.058 (0.143)
Government response -> Community mobility -> COVID-19 cases ^‡^	0.096 (0.084)	−0.334 (0.055) ***	-	0.224 (0.175)	−0.056 (0.106)	−0.228 (0.113) *	0.627 (0.241) **	0.088 (0.191)	0.073 (0.104)
*July–August (n = 62)*									
Community mobility -> COVID-19 cases	0.096 (0.131)	0.449 (0.065) ***	-	0.016 (0.205)	−0.040 (0.189)	−0.286 (0.104) **	−0.241 (0.106) *	−0.106 (0.130)	−0.488 (0.106) ***
Government response -> COVID-19 cases	−0.224 (0.105) *	0.045 (0.118)	-	−0.058 (0.177)	0.341 (0.145) *	−0.373 (0.153) *	-	0.209 (0.138)	0.199 (0.102)
Government response -> Community mobility	0.518 (0.087) ***	−0.025 (0.110)	−0.252 (0.124) *	0.404 (0.181) *	−0.643 (0.053) ***	0.250 (0.100) *	-	0.520 (0.073) ***	−0.629 (0.050) ***
Government response -> COVID-19 cases ^†^	−0.174 (0.118)	0.034 (0.133)	-	−0.052 (0.137)	0.367 (0.074) ***	−0.445 (0.147) **	-	0.154 (0.133)	0.506 (0.063) ***
Government response -> Community mobility -> COVID-19 cases ^‡^	0.050 (0.072)	−0.011 (0.052)	-	0.006 (0.100)	0.026 (0.125)	−0.071 (0.034) *	-	−0.055 (0.073)	0.307 (0.077) ***
*September–October (n = 61)*									
Community mobility -> COVID-19 cases	−0.173 (0.098)	−0.431 (0.128) **	-	−0.640 (0.116) ***	−0.620 (0.052) ***	−0.250 (0.112) *	−0.293 (0.097) **	−0.059 (0.150)	0.053 (0.168)
Government response -> COVID-19 cases	0.398 (0.081) ***	0.116 (0.141)	-	0.257 (0.125) *	0.468 (0.054) ***	−0.020 (0.135)	−0.416 (0.085) ***	0.207 (0.096) *	−0.331 (0.121) **
Government response -> Community mobility	0.387 (0.117) **	−0.527 (0.056) ***	0.628 (0.055) ***	−0.805 (0.036) ***	−0.442 (0.082) ***	0.347 (0.096) ***	0.416 (0.096) ***	−0.359 (0.125) **	0.462 (0.088) ***
Government response -> COVID-19 cases ^†^	0.331 (0.072) ***	0.344 (0.107) **	-	0.773 (0.041) ***	0.742 (0.052) ***	−0.107 (0.125)	−0.538 (0.073) ***	0.228 (0.101) *	−0.306 (0.082) ***
Government response -> Community mobility -> COVID-19 cases^‡^	−0.067 (0.052)	0.228 (0.074) **	-	0.515 (0.102) ***	0.274 (0.048) ***	−0.086 (0.046)	−0.122 (0.041) **	0.021 (0.062)	0.025 (0.079)
*November–December (n = 61)*									
Community mobility -> COVID-19 cases	0.232 (0.434)	0.504 (0.069) ***	-	0.270 (0.227)	−0.206 (0.178)	−0.385 (0.181) *	0.292 (0.119) *	0.043 (0.132)	0.037 (0.152)
Government response -> COVID-19 cases	0.308 (0.331)	0.552 (0.067) ***	-	−0.269 (0.215)	0.167 (0.168)	0.084 (0.164)	0.369 (0.162) *	0.544 (0.184) **	−0.258 (0.159)
Government response -> Community mobility	−0.687 (0.079) ***	0.188 (0.099)	−0.664 (0.064) ***	−0.817 (0.025) ***	−0.667 (0.053) ***	−0.740 (0.036) ***	0.614 (0.099) ***	−0.759 (0.063) ***	0.513 (0.062) ***
Government response -> COVID-19 cases ^†^	0.149 (0.101)	0.646 (0.069) ***	-	−0.490 (0.099) ***	0.305 (0.115) **	0.370 (0.104) ***	0.549 (0.145) ***	0.512 (0.157) **	−0.239 (0.144)
Government response -> Community mobility -> COVID-19 cases ^‡^	−0.159 (0.301)	0.095 (0.049)	-	−0.221 (0.186)	0.138 (0.122)	0.285 (0.133) *	0.180 (0.078) *	−0.033 (0.102)	0.019 (0.080)

^†^ Total effect; ^‡^ Indirect effect; * *p*-value ≤ 0.05; ** *p*-value ≤ 0.01; *** *p*-value < 0.001.

## Data Availability

Publicly available datasets were analyzed in this study. This data can be found here: www.bsg.ox.ac.uk/covidtracker (accessed on 10 January 2021), https://www.google.com/covid19/mobility/ (accessed on 11 January 2021), and https://ourworldindata.org/coronavirus (accessed on 11 January 2021).

## Supplementary Materials

The following supporting information can be downloaded at: https://www.mdpi.com/article/10.3390/healthcare10102003/s1, Table S1: Operational definition of the indicators included in government response.
