# Supplementary material for: Effects of the Government Response and Community Mobility on the COVID-19 Pandemic in Southeast Asia
Source: Healthcare (Basel). 2022 Oct 11;10(10):2003. doi: 10.3390/healthcare10102003 (PMC9602190; doi:10.3390/healthcare10102003)
Supplement: Supplementary file 1 [file healthcare-10-02003-s001.zip › healthcare-1907646-supplementary.pdf]

Table S1. Operational definition of the indicators included in government response

Code	Indicator	Definition	Measurement	Geographic scope/sectoral scope
Closures and containment				
C.1	Closing School	Data of the closings of schools and universities	0 - no measurements<br>1 - Closing advocate<br>2 - obligatory closure (some levels only, i.e., Secondary school only)<br>3 - compulsory closure of all stages<br>no information - blank	0 - specific<br>1 - universal<br>no information - blank
C.2	Closing workplace	Data of closings at workplaces	0 - no measurements<br>1 - Closing advocate (or work from home)<br>2 - For some industries or groups of employees, obligatory closure (or work from home)<br>3 - mandatory closure of all but necessary workplaces (or work from home) (i.e., grocery stores, doctors)<br>no information - blank	0 - specific<br>1 - universal<br>no information - blank
C.3	Public gatherings cancel	Data cancellation of public gatherings	0 - no measurements<br>1 – advocate cancel<br>2 - mandatory cancel<br>no information - blank	0 - specific<br>1 - universal<br>no information - blank
C.4	Limitations on gatherings	Data of restriction number of people on private gatherings	0 - no constraints<br>1 - limits on the selection of more than 1000 individuals<br>2 - Limits on meetings between 101-1000 individuals<br>3 - Limits on meetings between 11-100 individuals	0 - specific<br>1 - universal<br>no information - blank

			4 - limits on meetings with ten or fewer individuals<br>no information - blank	
C.5	Public transport closure	Public transport data closing	0 - no measurements<br>1 - advocate closing (or substantially reducing the quantity of available transport)<br>2 - obligatory closure (or banning it from being used by most citizens)<br>no information - blank	0 - specific<br>1 - universal<br>no information - blank
C.6	Stay at home policy	Data of stay at home policy	0 - no measurements<br>1 - advocate stay at home<br>2 - Compulsory homestay with exceptions for daily workouts, grocery shopping, and essential trips<br>3 - obligatory homestay with limited exceptions (i.e., allowed to leave only once a week)<br>no information - blank	0 - specific<br>1 - universal<br>no information - blank
C.7	Internal movement limitations	Data of internal movement limitation	0 - no measurements<br>1 - advocate not traveling between regions and cities<br>2 - internal limits on travel in place<br>no information - blank	0 - specific<br>1 - universal<br>no information - blank
C.8	International restrictions on travel	Data of international movement restrictions	0 - no measurements<br>1 - screening<br>2 - quarantine arrivals from regions at high risk<br>3 - restriction of arrivals from some areas	-

			4 - prohibition of all regions or absolute closing of borders<br>no information - blank	
Economic measures				
E.1	Support for income	The government's data protects the salary or gives the money in cash, general fundamental pays of individuals who lose their positions or can't work.	0 - no support for salaries<br>1 - The government replaces <50 percent of the missing wage<br>2- The government replaces roughly 50 percent of the missing wage<br>no information - blank	0 - formal sector workers only<br>1 – all sector workers<br>no information - blank
E.2	Debt or contract relief for houses	Data if the government is freezing economic obligations	0 - No<br>1 - relief limited to one form of contract<br>2 - Substantial debt or contract relief<br>no information - blank	-
Health measures				
H.1	Public campaigns for info	Data of involvement of public info campaigns	0 - No awareness program for the public<br>1 - information from public leaders<br>2 - organized campaign for public information (i.e., social media)<br>no information - blank	0 - specific<br>1 - universal<br>no information - blank
H.2	Testing regulation	Who can get checked for PCR?	0 - no testing regulation<br>1 - only those that have signs and fulfill particular requirements (i.e., returned from overseas, closed contact with confirmed case)<br>2 - examination of those displaying signs	-

			3 - public open testing, i.e., providing asymptomatic individuals with testing)<br>No information - blank	
H.3	Contact tracing	Are governments doing extensive tracing of contacts?	0 - no tracing of contacts<br>1 - minimal tracing of contacts<br>2 - extensive contact tracing<br>no information - blank	-
H.6	Coverings of face regulation	Data of public space policies on the use of face-covering	0 - no policy at all<br>1 - advocate<br>2 - mandatory outside of some specified shared/public spaces<br>3 - in all shared/public spaces, mandatory<br>4- mandatory at all times outside the home<br>no information - blank	0 - specific<br>1 - universal<br>no information - blank
H.7	Vaccination regulation	Data of vaccine delivery regulation	0 – not available<br>1 – Available for one of the leading workers/medically vulnerable/aging group<br>2 - Available for two of leading workers/medically vulnerable/aging group<br>3 - Available for all of the leading workers/medically vulnerable/aging group<br>4 - Available for all three groups plus partial additional availability (wide group/age selection)<br>5 – Available for all<br>no information - blank	0 – Paid by individual<br>1- Paid by government or subsidized<br>no information - blank

The calculation of each indicator (sub-index scores) use the formula:

$$I_{j,t} = 100\frac{v_{j,t} - 0.5(F_{j-}f_{j,t})}{N_j}$$

Remarks:

I = score sub-index

j = any indicator given

t = any day given

$v_{j,t}$ = on the ordinal scale, the recorded policy value

F_j = if that indicator does have a flag (Fj=1 if the indicator has a flag variable, or 0 if there is no flag variable on the indicator)

$f_{j,t}$ = if that indicator does have a flag, the recorded binary flag to this indicator

N_j = the indicator's highest value

The indexes are used:

a. Containment index: indicators C.1, C.2, C.3, C.4, C.5, C.6, C.7, C.8
b. Health index: indicators H.1, H.2, H.3, H.6, H.7
c. Economic support index: indicators E.1, E.2

The calculation of each index use formula:

$$Index = \frac{1}{k}\sum_{j=1}^{k} I_j$$

Remarks:

k = the number of indicators of the components in the index

j = any indicator given

I_j = the sub-index score for an individual indicator
